# Micronized Calcite Foliar Treatments as an Approach to Enhancing Yield and Quality Parameters of Red Globe Grape (*Vitis vinifera* L.) Under Semi-Arid Conditions

**DOI:** 10.3390/plants15132013

**Published:** 2026-06-29

**Authors:** Tuba Uzun Bayraktar

**Affiliations:** Department of Horticulture, Faculty of Agriculture, Siirt University, Siirt 56100, Türkiye; tubauzun@siirt.edu.tr; Tel.: +90-5422272137

**Keywords:** *Vitis vinifera*, Red Globe, foliar biostimulant, micronized calcite, viticulture, semi-arid, yield and quality

## Abstract

**Background**: Foliar fertilization is a crucial practice in modern viticulture to enhance grape yield and fruit quality. Micronized calcite is a fine-particle mineral fertilizer that potentially improves vine performance; however, its treatment timing and optimal dosage require further scientific validation under field conditions. **Methods**: This study investigated the effects of the use of micronized calcite as a foliar biostimulant on the Red Globe grape variety. Control and three treatments were compared: a control (no treatment), a single treatment (pre-bloom, 0.5%), two treatments (pre- and post-bloom, 0.5% + 0.5%), and three treatments (pre-bloom, post-bloom, and véraison, 0.5% + 0.5% + 0.5%). The evaluation encompassed phenological stages, total effective temperature, yield components, cluster and berry characteristics, and must composition. This was a single-season trial (2022) conducted at a single location with three replicates per treatment (*n* = 3); multi-year, multi-location validation is therefore required before the findings can be generalized. **Results**: All micronized calcite treatments slightly shortened the vegetation period compared to the control. Under the conditions of this single-season trial, the single pre-bloom treatment was associated with the highest yield parameters, with the average number of clusters per vine, cluster weight, and total grape yield per vine being higher than the control by 48.67%, 51.16%, and 121%, respectively. For must composition, only must yield differed significantly (between the 1st and 3rd treatments; *p* < 0.05); soluble solids content (+5.64%) and ripening index (+16.99%) were numerically higher but not statistically significant (*p* ≥ 0.05) and are therefore reported as trends rather than improvements. By contrast, the two-treatment (pre- and post-bloom) showed the highest values for physical berry traits, with cluster width and 100-berry weight exceeding the control by 35.03% and 11.11%, respectively. **Conclusion**: Under the semi-arid conditions of this single-season trial, foliar treatments of micronized calcite, particularly a single pre-bloom application, were associated with notable improvements in yield and must quality of Red Globe grapevines. These preliminary findings suggest that finely milled calcite may serve as a promising supplementary foliar fertilizer in viticulture; however, the results are context-specific, and multi-year, multi-location trials are required before broader recommendations can be made.

## 1. Introduction

Modern viticulture faces significant challenges, including the adverse effects of climate change, soil degradation, and the environmental pollution caused by the excessive use of conventional chemical fertilizers. To maintain sustainable yield and fruit quality under these increasingly stressful conditions, there is a growing need for environmentally friendly and effective agricultural practices. In this context, the application of fine-particle foliar fertilizers has emerged as a promising and sustainable strategy to rapidly enhance plant nutrition, improve stress tolerance, and reduce the dependency on intensive soil-applied chemicals.

Income from viticulture can be enhanced by increasing grape yield per unit area [1]. As it was in the other plant production activities, fertilization is an essential method frequently used by producers to increase yield and quality in viticulture [2,3]. Although different cultural practices (winter pruning, shoot thinning, tip removal, bark ringing, leaf thinning, etc.) are carried out to increase yields in viticulture, fertilization has a special importance [4,5,6,7]. Fertilizer dose, composition, application method and period directly affect the grape yield and quality [8,9,10,11,12].

Grapevines need various nutrients throughout different growing stages [13]. Ideally developing grapevine shoots uptake macronutrients (less than 0.1% of dry weight) and micronutrients (less than 100 ppm) from the soils [14]. Insufficient uptake of iron (Fe), boron (B), zinc (Zn) and copper (Cu) may result in some physiological disorders [15,16].

Molybdenum (Mo) deficiency may reduce the number of berries in a cluster and result in shot berries (through affecting development of the pollen tube) [17,18]. In addition, in vineyards with severe B deficiency, leaves become smaller, leaf edges become curled downwards and fall off [19,20]. B and Mo deficiency can be eliminated through fertilization if detected in dormant season. However, if such a deficiency is detected during the vegetative period, foliar fertilization should be preferred for faster and more effective results [16]. Previous research on some grape varieties (*V. vinifera* L.) showed that foliar application of B and Mo increased grape yield and quality [18,21].

In recent years, the development of fine-particle and micronized fertilizers has gained significant attention in agricultural production. Micronized fertilizers, due to their reduced particle size, are designed to improve nutrient use efficiency, enhance leaf penetration, and increase overall plant yields [22]. They also have the potential to reduce soil pollution and other environmental risks commonly associated with excessive chemical soil fertilization [23]. Furthermore, advanced foliar fertilizers are formulated to combine plant nutrition and plant protection. By creating a microscopic homogeneous layer on the leaf surface, these products aim to provide a physical barrier alongside nutrient supplementation. Under Regulation (EU) 2019/1009, fertilizing products supply nutrients, whereas plant biostimulants act by stimulating natural plant processes independently of nutrient content [24]. Because the foliar uptake of calcium from calcite is physiologically limited, we treat micronized calcite here as a chemically inert, fine-particle mineral film with biostimulant-type effects rather than as a calcium fertilizer. Empirical studies of such films report that they form a homogeneous leaf surface layer, modify the leaf microclimate, and can influence gas exchange and photosynthetic performance, contributing to yield and quality under high-radiation conditions [25,26,27,28].

Akçay and Akın [29] investigated the effects of different amounts of leaf removal and micronized calcite foliar fertilizer applications on the yield and quality of the Sultani Çekirdeksiz grape variety. They recommended high leaf removal combined with potassium humate and micronized calcite treatments to increase the yield. Similarly, Şimşek Gözlemeci [30] evaluated the effects of micronized calcite treatments on the vegetative development of vine saplings in various grafted rootstock–scion combinations. The study reported that micronized calcite treatments had positive effects on vegetative development and mineral nutrition, increasing Ca accumulation in tissues and positively affecting shoot development levels.

With recent developments in fine-milling and micronization technologies, fertilizer companies have introduced several micronized mineral fertilizers to the market. However, despite the growing body of literature on Herbagreen-type products, critical knowledge gaps remain regarding the optimal application timing and frequency of micronized calcite under field conditions, particularly in semi-arid viticultural regions. Most existing studies have focused on vegetative development or isolated yield components, without systematically comparing phenological-stage-specific application strategies across a comprehensive set of yield, quality, berry, and color parameters, complemented by descriptive characterization of leaf nutrient status. Furthermore, the Red Globe variety (one of the most commercially important table grape cultivars) has received limited attention in this context. Therefore, this study was designed to address these gaps by investigating the effects of micronized calcite (Multigreen = Herbagreen) applied at increasing numbers of applications across distinct phenological stages (pre-bloom only; pre- and post-bloom; pre-bloom, post-bloom, and véraison) on the yield, fruit quality, and berry color parameters of Red Globe grapevines, with leaf nutrient profile reported descriptively as a complementary characterization, under the semi-arid ecological conditions of Siirt province, Türkiye. It is important to clarify the premise of this study: micronized calcite is not applied here to correct a calcium deficiency. Foliar calcium uptake from calcite is physiologically limited, and the soil at the experimental site is calcium-rich. Rather, the product is evaluated as a chemically inert, fine-particle mineral film whose hypothesized effects are physical and stress-mitigating. These effects include modifying the leaf microclimate, increasing leaf surface reflectance, and reducing heat and transpirational stress under semi-arid conditions, with possible biostimulant-type responses documented for kaolin and analogous particle films in grapevine. In contrast to previous studies, which have largely focused on a single application timing or on vegetative development [5,29,30,31], the present work systematically compares phenological-stage-specific application strategies across yield, fruit quality, berry, and color parameters in the commercially important Red Globe cultivar.

## 2. Results and Discussion

Phenological observations: Budburst in all vines took place in the first week of April in the vegetation year 2022. While full bloom took place in the second week of May in the control treatment, in the other treatments full bloom was observed three days earlier following the first micronized calcite treatment before flowering (28.04.2022). The véraison stage occurred in the first week of July in the control treatment, in the last week of June in the 1st treatment, and following the second micronized calcite treatment after flowering (25.05.2022) in the third week of June in the 2nd and 3rd treatments, six days earlier than the control and four days earlier than the 1st treatment. Since phenological stages were recorded as single observations rather than replicated measurements, these dates are reported descriptively and were not subjected to statistical comparison. It was indicated in previous studies on different products, micronized calcite (Multigreen = Herbagreen) treatments at different periods and in different doses shortened the vegetation period and provided earliness [32,33,34,35].

Total effective temperature (TET): Total effective temperature is the most important parameter taken into consideration when determining whether viticulture will be done in a region or which varieties will be grown in that ecology.

The TET value from budburst to harvest was calculated as 2233.91 degree-day (dd) for all treatments. Between full bloom and véraison, following the first micronized calcite treatments before flowering, the TET value was calculated as 783.98 dd, while in the 2nd and 3rd treatments TET reached 703.96 dd over a shorter interval. One possible interpretation, consistent with previous reports on foliar applications of macro- and micronutrients [36,37], is that the macro- and micronutrient content of micronized calcite may have contributed to earlier completion of vegetative stages; however, other explanations cannot be excluded from the present dataset, including a physical particle film effect on leaf microclimate and inter-vine variation under non-replicated phenological observation (Table 1).

The shortening of the vegetation period between full bloom and véraison in treated vines, observed under the high-temperature conditions of the 2022 growing season, suggests that the pre-bloom micronized calcite treatment may have supported more efficient reproductive development under the prevailing thermal regime.

Solar radiation (W/m^2^): Sunlight has a great impact on viticulture. Solar radiation designates several characteristics of grapes, such as sugar, acidity, color, aroma and ripening. Plants need a certain amount of light to perform maximum photosynthesis. Since light provides all the energy required for photosynthesis, it is the most important factor affecting net assimilation. In order for plants to use chemical bond energy in the production of organic matter, that is, in photosynthesis, the plant must block the light by its canopy and the ability to convert this blocked light energy into chemical energy, that is, its light use efficiency, must be high [38].

Solar radiation from budburst to harvest was determined as 124.81 W/m^2^ for all treatments. Between full bloom and véraison, following the first micronized calcite treatments before flowering, vegetation period was prolonged and solar radiation was determined as 95.83 W/m^2^. The other two micronized calcite treatments extended vegetation period as compared to control and shortened as compared to the first treatments and solar radiation was then calculated as 95.25 W/m^2^. It is because biostimulant of micronized calcite promoted growth in the period until the flowering phase, when vegetative growth accelerated, but it lost this effect as generative growth accelerated after flowering (Table 1).

The higher solar radiation exposure recorded in the 1st treatment group during the full bloom to véraison period, compared to the 2nd and 3rd treatments, may have contributed to the superior yield and must quality parameters observed in this treatment, as adequate light interception during this critical window is known to promote sugar accumulation and berry development.

**Figure 1:**
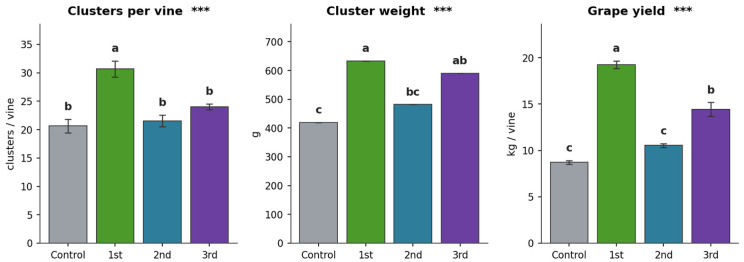
Effects of micronized calcite treatments on yield parameters: clusters per vine, cluster weight (g), and grape yield per vine (kg). Bars are means ± standard error (*n* = 3); different letters indicate significant differences among treatments (Tukey HSD); *** *p* < 0.001.

Effects of experimental treatments on yield parameters: The differences in all parameters of the experimental treatments were found to be significant (Figure 1). The greatest average number of clusters (30.67 clusters/vine), cluster weight (633.75 g) and grape yield (19.26 kg/vine) were obtained from the 1st treatments.

The superior performance of the 1st treatment in yield parameters, despite receiving the lowest total dose of micronized calcite, suggests that application timing is a more decisive factor than cumulative dose in determining treatment efficacy.

In a study conducted on Shiraz grape variety, in terms of cluster weight and grape yield per vine, application before flowering was indicated as the most suitable time for nanotechnological foliar fertilizer applications used at different periods and in different doses [39]. Harris, Kriedemann, and Possingham [40] supports the finding that the completion of formation of pericarp tissue in grape berries may begin 5–10 days before flowering and continue until approximately 25 days after pollination. It was indicated in a study conducted on different levels of leaf removal and foliar fertilizer applications in Sultani Çekirdeksiz grape variety that the highest grape yield (22.30 kg/vine) was obtained from the “heavy leaf removal + potassium humate + micronized calcite” treatments [29]. In a study where some organic plant growth regulators were applied to the Narince grape variety, the highest grape yield (7.1 kg/vine) was obtained from Herbagreen treatments. It was also indicated that the highest cluster weight (294.4 g) was obtained from Herbagreen treatments [41]. It was stated in a study conducted on Horoz Karası and Gök Üzümü varieties that Herbagreen treatments did not have any significant effects on cluster weight of Horoz Karası variety, while it increased cluster weight of Gök Üzümü variety as compared to control treatments [5]. Researchers who examined the effects of various foliar fertilizers on cluster characteristics of grape varieties reported that they obtained different results. Researchers have reported that a variety grown in different ecologies may respond differently to the applied fertilizer and that different varieties grown in the same ecology may also show quite different responses [18,42,43,44]. In previous studies on both grapes and other products, it was stated that micronized calcite (Multigreen = Herbagreen) treatments in different periods and at different doses had positive effects on yield parameters [5,31,34,45,46,47,48]. Present findings comply with those earlier ones.

Effects of experimental treatments on cluster, berry and leaf parameters: Among the parameters examined in this group, only cluster width and average leaf weight differed significantly among treatments (*p* < 0.05; Table 2). The 2nd treatment was associated with significantly greater cluster width (19.89 cm) than the control (14.73 cm), and the 3rd treatment with significantly greater average leaf weight (4.06 g) than the control (2.48 g). For the remaining parameters (cluster length, berry width, berry length, 100-berry weight, number of seeds per berry, average seed weight, and leaf chlorophyll content), no statistically significant differences were detected among treatments (*p* ≥ 0.05). Although numerical differences in these parameters are reported in Table 2 for completeness (e.g., the highest values were obtained from the 2nd treatment for most physical berry traits and from the 1st treatment for leaf chlorophyll), these differences should not be interpreted as evidence of treatment effects.

Current literature demonstrates that micronized calcite and similar foliar fertilizer applications significantly improve the physical characteristics of clusters and berries in grapevines. Studies conducted on various grape varieties (such as Shiraz, Sultani Çekirdeksiz, Horoz Karası, Gök Üzümü, Alphonse Lavellée, and Narince) have reported that micronized calcite (Herbagreen/Multigreen) treatments provide statistically significant increases in cluster width and length, as well as berry dimensions including width, length, and weight [5,29,31,39,41]. These findings are consistent with previous research indicating that foliar fertilizers containing macro- and micronutrients increase berry weight when applied at the correct phenological stages and in specific doses [8,11,18,21,42,43]. In the present study, although the 2nd treatment showed numerically higher values for most physical cluster and berry traits of the Red Globe grape variety, these differences, except for cluster width and average leaf weight, did not reach statistical significance (*p* ≥ 0.05) under the present design; treatment effects on these parameters therefore cannot be concluded from this dataset, and the values are reported for completeness only.

In addition to their positive effects on fruit quality, these treatments are known to play a stimulating role in vegetative development and leaf physiology. It has been reported that foliar-applied nutrients (especially K, Mg, and Ca) exhibit a positive correlation with leaf chlorophyll content (SPAD values) and contribute to the development of a healthy, green canopy structure required for optimum photosynthesis [36,49]. Furthermore, recent studies on various grapevine saplings have confirmed that micronized calcite treatments significantly increased chlorophyll contents, fresh leaf weights, and overall biomass, suggesting a growth-promoting role in vine development [30,31,50].

Notably, while the 1st treatment was associated with the highest yield components, the 2nd treatment was associated with the highest values for physical berry dimensions. One possible interpretation is that post-bloom timing coincides with the cell division and expansion phases of berry development, which in *Vitis vinifera* are reported to extend from approximately 5–10 days before flowering to about 25 days after pollination [40]. Because we did not assess cytological or biochemical markers of these phases, we present this as a working hypothesis rather than an established mechanism. The results nonetheless fit the broader principle that aligning foliar applications with specific developmental stages can influence the magnitude of the response, a relationship that future trials could test directly.

**Figure 2:**
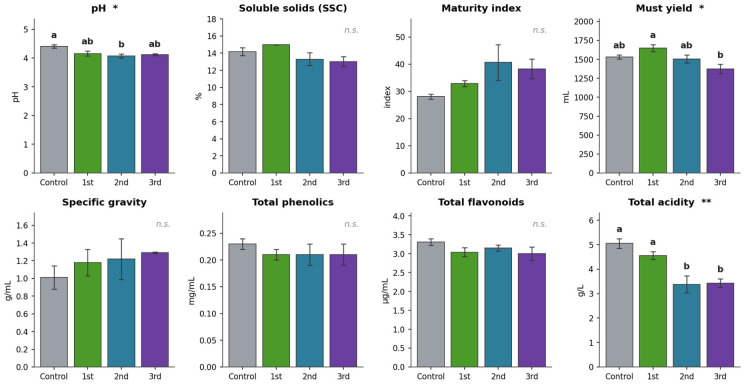
Effects of micronized calcite treatments on grape juice (must) composition. Bars are means ± standard error (*n* = 3); different letters indicate significant differences among treatments (Tukey HSD); * *p* < 0.05, ** *p* < 0.01; n.s. = not significant. Total phenolics (mg/mL), gallic acid equivalent; total flavonoids (µg/mL), rutine equivalent; total acidity (g/L), tartaric acid equivalent.

Effects of experimental treatments on must parameters: Among the must parameters examined, statistically significant differences among treatments (*p* < 0.05) were detected only for pH, must yield, and total acidity (Figure 2). The control treatment showed significantly higher pH (4.41) and total acidity (5.05 g/L) than the 2nd and 3rd treatments, while the 1st treatment showed significantly greater must yield (1647.50 mL) than the 3rd treatment. For the remaining parameters (soluble solids content (SSC), maturity index, specific gravity, total phenolics, and total flavonoids), no statistically significant differences were detected among treatments (*p* ≥ 0.05). Although mean values for these parameters are presented in Figure 2 to allow full reporting of the dataset (e.g., numerically higher SSC under the 1st treatment and numerically higher maturity index under the 2nd treatment), they should not be interpreted as evidence of treatment effect.

The specific gravity values observed in the present study (1.01–1.29) were somewhat higher than those typically expected for clarified grape juices at the corresponding soluble solids range, in which values around 1.05–1.06 are commonly reported [51]. Such deviations are not uncommon in pycnometric measurements of grape must and may reflect the contribution of soluble non-sugar constituents (organic acids, tartrate salts, polyphenols, and minerals) that add to mass without being captured by refractometric Brix measurement, as well as small temperature and calibration variations inherent to gravimetric pycnometry [51,52]. Importantly, specific gravity did not differ significantly among the experimental treatments (*p* ≥ 0.05; Figure 2) and is therefore reported here for completeness rather than as evidence of a treatment effect. The maturity index values observed in the present study (28.14–40.69) reflect the calculation convention used here, in which titratable acidity is expressed in g/100 mL rather than g/L; expressed as the more common SSC/total acidity (g/L) ratio, these values would correspond to approximately 2.81–4.06, which is within the range typically reported for table grapes at harvest. Furthermore, the relatively high maturity index values are consistent with the rapid degradation of organic acids characteristic of *Vitis vinifera* L. ripening under hot, semi-arid conditions, where high temperatures during the maturation period (Table 3; June–August mean temperatures of 28–33 °C) accelerate malic acid catabolism and shift the sugar-to-acid balance toward higher index values. Similar patterns of elevated maturity indices have been reported for table grape cultivars grown in Mediterranean and continental semi-arid climates of Türkiye and neighboring regions [5,29,53].

Koç [53] applied seaweeds to Cabernet Sauvignon grape variety at different times and in different doses and indicated that seaweed treatments had significant effects on must pH, SSC and total acidity values (*p* < 0.05). The 2nd period (3.73) had greater pH value than the 1st period (3.59). In terms of SSC, again the 2nd period (24.88%) had a greater value than the 1st period (24.37%). For total acidity, 2nd period (10.73 g/L) had a lower value than the 1st period (11.41 g/L). Odabaşıoğlu, Adıgüzel, and Gürsöz [39] indicated that different fertilizer doses and application periods did not significantly change must dry matter (25.4%), tartaric acid content (6.02 g/L) and harvest time of Shiraz grape variety, but the greatest values were obtained from the treatments performed before flowering. Akçay and Akın [29] applied different leaf removal rates and foliar fertilizers to Sultani Çekirdeksiz grape variety and indicated that ripening index increased and must yield decreased with the experimental treatments. Akın [5] conducted a study on Horoz Karası and Gök Üzümü varieties and reported that Herbagreen treatments reduced dry matter content and increased ripening index of both grape varieties. It was reported in another study conducted on Alphonse Lavellée grape variety that micronized calcite treatments reduced dry matter content and pH, but increased total acidity [31]. Dilek [41] applied some organic plant growth regulators to Narince grape variety and obtained the highest dry matter content (19.3%) and pH value (3.83) from Herbagreen treatments. It was also determined that Herbagreen treatments reduced titratable acidity and increased must yield as compared to the control.

The higher SSC and ripening index values in the 1st treatment, combined with its higher yield, may indicate that a single pre-bloom application is associated with a more favorable balance between vegetative and reproductive resource allocation than schemes with multiple applications. Although altered source–sink dynamics offer one plausible interpretation, this hypothesis was not directly tested in the present study and would require dedicated carbon allocation or biomass partitioning measurements for confirmation.

The improvements in yield parameters and must quality observed under the single pre-bloom treatment are consistent with a positive plant response to micronized calcite under the conditions of the present study. However, the underlying mechanisms of this response require careful interpretation. Calcium carbonate (CaCO_3_), the principal active constituent of micronized calcite, has low aqueous solubility, and foliar uptake of calcium is generally considered limited owing to its restricted phloem mobility and the structural barriers presented by the leaf cuticle [54,55]. Accordingly, the responses observed in the present trial are unlikely to be attributable solely to a direct nutritional contribution of foliarly applied calcium. Rather, multiple, non-mutually exclusive mechanisms can plausibly account for the observed effects, and these are best considered jointly rather than in isolation.

First, fine-particle mineral applications are reported to form a microscopic, homogeneous film on the leaf surface that can optimize the leaf microclimate, increase albedo (light reflectance) and reduce leaf temperature and transpirational stress under high-radiation, semi-arid conditions [25,26,27,28]. Such particle film effects have been documented for kaolin and analogous mineral products in grapevine and other fruit crops [26,56] and are particularly relevant to the climatic regime recorded at the experimental site, where summer temperatures regularly exceeded 40 °C and solar radiation peaked above 1000 W/m^2^ (Table 3). Second, even modest incremental delivery of calcium and trace elements through the leaf surface, though quantitatively limited, may contribute to cell wall stabilization through pectin cross-linking, membrane integrity, and calcium-mediated signaling, all of which are well-established roles of calcium in fruit development [54,57]. Third, the broader stimulatory effects reported here are consistent with a generalized biostimulant response previously documented for foliar calcium and mineral-based products in fruit crops and grapevines [58,59,60,61,62,63].

Importantly, none of these candidate mechanisms (physical microclimate modification, marginal nutritional contribution, or generalized biostimulation) was directly measured in the present study, and the relative contribution of each cannot be resolved from the present dataset. Definitive mechanistic attribution would require dedicated investigations including, for example, leaf level gas exchange and chlorophyll fluorescence measurements under controlled radiation conditions, isotope tracer studies of foliar Ca uptake and translocation, and direct measurement of leaf surface temperature and reflectance following treatment. These represent priority directions for future research and are essential before the present field-level associations can be interpreted as evidence of any single physiological pathway.

Effects of experimental treatments on berry color parameters: Color parameters were measured at harvest period with the use of a colorimeter (Pen color art 1L model, Artoksi MSM, Istanbul, TR) and Hunter color measuring system (L*, a*, b*) [64]. Furthermore, chroma and hue angles were calculated using these values [65].

**Figure 3:**
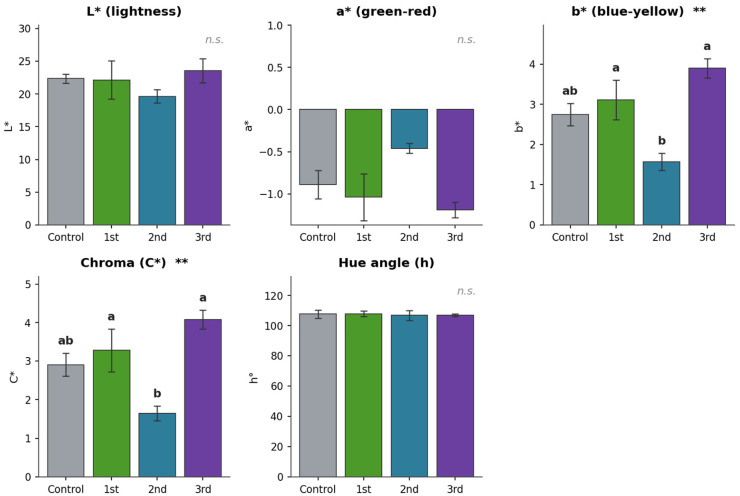
Effects of micronized calcite treatments on berry color parameters (CIELAB L*, a*, b*, chroma C*, and hue angle h°). Bars are means ± standard error (*n* = 3); different letters indicate significant differences among treatments (Tukey HSD); ** *p* < 0.01; n.s. = not significant. L = 0 black (dark), L = 100 white (light); a = +60 red, a = −60 green; b = +60 yellow, b = −60 blue.

The greatest L* (23.56), b* (3.90) and chroma (4.08) values were obtained from the 3rd treatments. The greatest a* value (−0.46) was obtained from the 2nd treatments and the greatest hue angle (h° = 107.91) value was obtained from the 1st treatments (Figure 3). There were significant differences in b* and chroma (C*) values of the experimental treatments (Figure 3). The lower the L* value, the darker the berry color becomes.

While the effects of experimental treatments on color values L*, a*, b* and chroma (C*) of Sultani Çekirdeksiz grape variety were found to be insignificant, they had significant effects on hue angle (h°) values (*p* > 0.05) [66]. Akçay and Akın [29] applied different leaf removal rates and foliar fertilizers to Sultani Çekirdeksiz grape variety and obtained the highest L* value from Low leaf removal + Micronized Calcite treatments and the highest a* and b* values from the control treatments. In Horoz Karası variety, which is a colorful grape variety like Red Globe, contrary to Gök variety, Herbagreen treatments decreased L* values and increased a* and b* values as compared to the control [5].

Among the color parameters examined, statistically significant differences among treatments were detected only for b* and chroma (C*) values (*p* < 0.01; Figure 3), while L*, a*, and hue angle (h°) did not differ significantly. Significant differences appeared for b* and chroma but not for the other color indices. This selective pattern may indicate that treatment timing affects specific pigment-related parameters rather than color as a whole. Because we did not analyze the underlying biochemical pathways (for example, anthocyanin or carotenoid composition), this explanation remains tentative and should be tested directly in future work.

Leaf nutrient profile under the experimental treatments: Mean values of leaf macro- and micronutrient contents under each treatment are presented in Table 4. Because leaf nutrient analyses in the present study were intended as a complementary characterization of the treatments rather than as a primary endpoint, these data were nonetheless subjected to one-way ANOVA followed by Tukey’s HSD test (α = 0.05); no statistically significant differences among treatments were detected for any leaf nutrient (*p* ≥ 0.05; Table 4). Numerical values across all treatments fell within ranges generally considered sufficient for *Vitis vinifera* L. according to standard references [67], and no marked deviations from the control profile were apparent. Consistent with this, one-way ANOVA confirmed that none of the leaf nutrients differed significantly among treatments (*p* ≥ 0.05; Table 4), indicating that the treatments did not measurably alter leaf nutritional status under the conditions of this single-season trial.

Sabir et al. [68] applied nanotechnological fertilizers alone or in combinations and reported positive effects on leaf nutrients as compared to control grapes. It was stated that nanotechnological fertilizer treatments resulted in sufficient N and high P and K levels in leaves of Tarsus Beyazı grape variety, but the differences from the control treatments were not found to be significant. Ca, Mg, Zn, Fe and Cu were found to be higher than the limiting values in the control and treatment groups. In terms of Mn content, the control group had higher values than the treatment groups, but differences were not found to be significant [69]. Dilek [41] conducted a study on Narince grape variety and obtained the highest Ca, Mg, Fe, Zn, B and Mn contents from Herbagreen treatments. Although these previous studies provide useful context regarding the potential influence of foliar treatments on grapevine leaf nutrient status, in the present study one-way ANOVA revealed no significant differences in leaf nutrient contents among treatments (*p* ≥ 0.05), consistent with the limited foliar uptake expected for a calcite particle film and indicating that the agronomic responses observed were not accompanied by detectable shifts in leaf nutritional status.

The climatic data recorded at the experimental vineyard (Table 3) provide an important context for interpreting the patterns of treatment response observed in the present study. The early phenological stages of the vine (budburst, full bloom, and the onset of berry development) coincided with April–May, when monthly mean temperatures rose from 17.9 °C to 24.2 °C and mean solar radiation reached its annual peak (278.06 W/m^2^ in April). The pre-bloom application of micronized calcite (28 April 2022) was therefore performed at a phenological stage in which leaf surfaces were rapidly expanding and were exposed to high-radiation loads under still-moderate temperatures, conditions under which the formation of a uniform mineral particle film on the leaf surface is most likely to be effective in modulating the leaf microclimate [25,26,54,55]. The subsequent reproductive and ripening phases (June–August) were characterized by progressively increasing temperatures (28.3–32.7 °C monthly means; daily maxima frequently exceeding 44 °C) combined with sharply declining relative humidity (down to monthly means of 25.1–26.9%), conditions known to impose substantial heat and water-related stress on grapevines and to accelerate organic acid degradation in ripening berries, as also reflected in the elevated maturity index values discussed above. These environmental constraints are consistent with the relatively low SSC values (13.0–15.0%) and high maturity indices observed across all treatments, and they suggest that the agronomic responses reported here should be interpreted as outcomes of micronized calcite application under heat- and radiation-stressed semi-arid conditions, rather than as generalizable responses across viticultural environments. The greater relative effectiveness of the single pre-bloom application, performed before the onset of the most stressful summer period, is also broadly consistent with the principle that early-season foliar interventions can pre-condition the leaf canopy for subsequent stress exposure, although direct measurements of leaf temperature, gas exchange, or water use efficiency would be required to substantiate this mechanism in the present cultivar and site.

Beyond the treatment-specific differences in individual parameters, several recurring patterns across the dataset can be examined as potential trade-offs between yield components and must quality. Although the limited replication of the present study precludes a formal correlation analysis with adequate statistical power, a qualitative inspection of the treatment means is informative. The 2nd treatment, which was associated with the largest cluster width and 100-berry weight, also exhibited the lowest soluble solids content (13.30%) and the highest maturity index of all treatments, a combination consistent with the well-documented dilution of soluble solids accompanying increased berry expansion in *Vitis vinifera* L. [40]. Conversely, the 1st treatment displayed an apparent decoupling of this trade-off, combining the highest yield per vine with the highest SSC, suggesting that pre-bloom application may support both reproductive load and concurrent sugar accumulation more efficiently than later or repeated applications, although it is unclear whether this reflects a genuine physiological balance or sampling variability under low replication. A further pattern is the inverse association between yield-related components and total acidity: the control retained the highest acidity (5.05 g/L) together with the lowest yield, whereas all calcite-treated vines showed lower acidity together with higher yield, in line with the canonical fruit load effect on acid concentration. Finally, berry color parameters appeared largely independent of yield and SSC trends: the 3rd treatment produced the most pronounced b* and chroma values despite intermediate yield and the lowest SSC, indicating that pigment-related metabolism may respond to a partly distinct set of physiological signals. We therefore present these patterns as candidate trade-offs rather than firm conclusions; they mark balance points worth dedicated study in future trials with larger replication, multi-year coverage, and direct measurement of canopy carbon allocation, sugar and acid metabolism, and pigment biosynthesis.

Taken together, the results across the measured parameters point to a treatment-specific pattern within the present single-season dataset: the single pre-bloom application (1st treatment) was associated with the highest values for yield and must quality, the double application (2nd treatment) for physical berry and cluster dimensions, and the triple application (3rd treatment) for certain color parameters. This pattern may reflect the influence of application timing and frequency on distinct developmental windows of the vine, although the underlying physiological pathways were not directly assessed in this study and warrant dedicated investigation. While these observations may offer preliminary practical orientation for viticulturists tailoring foliar fertilization strategies under semi-arid conditions similar to those of Siirt province, their generalizability remains to be confirmed through multi-year and multi-location validation.

## 3. Materials and Methods

### 3.1. Plant Material and Experimental Site

**Figure 4:**
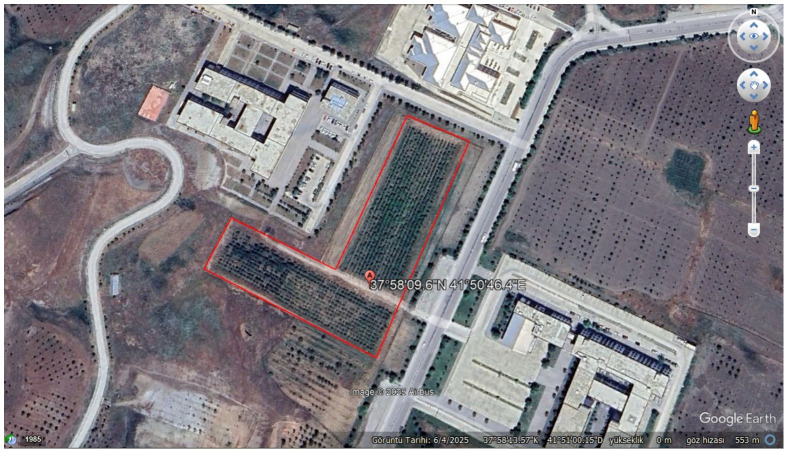
Satellite image of experimental vineyard [70].

Characteristics of experimental vineyard: Experiments were conducted at a vineyard located at Viticulture Research and Experiment Station of Siirt University Kezer Campus (37°58′9.6″ N latitude and 41°50′46.4″ E longitudes, 583 m altitude). Experimental vineyard was established with Red Globe grape variety grafted on 1103 Paulsen rootstocks in 2014. Vines were trained on a pergola system and planted at a 3 m × 1.5 m spacing (Figure 4).

Climate data for experimental vineyard: Climate data (temperature, relative humidity and solar radiation) of the experimental vineyard (from budburst till senescence) were recorded with a data logger (HOBO U12-013, Onset Computer Corporation, Bourne, MA (Massachusetts), USA) (1 record in 60 min) attached to the level of the tilting wire of the vine branches. The resultant data were used to calculate effective temperature totals (ETTs). Solar radiation was recorded in W/m^2^ with Apogee brand Pyranometer device connected to HOBO device and the resultant data (1 record in 60 min) was turned into monthly averages. The relevance of experimental treatments to climate parameters was also examined.

Climate data of the experimental vineyard were taken in 2022 vegetation year. From budburst until abscission, monthly average temperature, minimum temperature, maximum temperature, average relative humidity, minimum relative humidity, maximum relative humidity, average solar radiation, minimum solar radiation and maximum solar radiation were calculated and given in Table 3.

Budburst of Red Globe grapes took place on 05.04.2022 and the latest leaf fall date took place on 25.11.2022. Therefore, average temperature, relative humidity and solar radiation values were calculated using these dates. The highest average temperature (33 °C) and the lowest average temperature (12 °C) values were recorded in July and November, respectively. While the minimum temperature (0.3 °C) occurred in November, the maximum temperature (46 °C) occurred in July. The highest average relative humidity (72%) and the lowest average relative humidity (25%) values were recorded in November and July, respectively. While the minimum relative humidity (5.86%) occurred in June, the maximum relative humidity (100%) occurred in November. Since there was no solar radiation at nighttime, night data were not taken into consideration while calculating monthly average solar radiation and minimum solar radiation quantities. The highest monthly average solar radiation (278.06 W/m^2^) and the lowest monthly average solar radiation (63.19 W/m^2^) values were recorded in April and October, respectively. While the minimum solar radiation was measured as 3.05 W/m^2^ in all months, the maximum solar radiation was measured as 1001.20 W/m^2^ in April. Since the maximum solar radiation was taken into account, with the budburst of the vines on 05.04.2022, solar radiation values started to decrease with the progress of vegetative development.

These climatic characteristics, particularly the high summer temperatures and elevated solar radiation recorded during the critical reproductive stages of the vine (May–July), represent conditions under which foliar applications of fine-particle mineral products may exert their most pronounced effects by modulating the leaf microclimate and supporting nutrient availability during key developmental windows.

Soil characteristics of the experimental vineyard: Soil analysis was carried out at the laboratory of Siirt University Science and Technology Application and Research Center. Composite soil samples were taken from the 30–60 cm horizon, which represents the principal feeder root zone of the mature, deep-rooted vines at this site below the tilled surface layer [16,71]. Soil physical properties, macro and micronutrients were analyzed in accordance with Aksu [72]. Experimental soils were clayey (C) in texture, unsaline, highly limy, moderately alkaline with highly low organic matter content (Table 5). Organic matter was determined using the modified Walkley–Black wet oxidation method; the low value (0.92%) is typical of semi-arid clay soils. The reported organic matter value was obtained by converting the Walkley–Black organic-carbon result to organic matter using the conventional Van Bemmelen factor of 1.724 (organic matter = organic carbon × 1.724). Available phosphorus was determined using the Olsen sodium-bicarbonate method (TS ISO 11263) [73] and exchangeable potassium using the ammonium acetate method, both expressed in kg/da (the standard reporting unit of Turkish soil fertility analysis) and classified as “very low” and “sufficient”, respectively. Soil nitrogen was not included in this routine fertility analysis; nitrogen was instead supplied uniformly to all treatments through the basal fertigation described in Section 3.2. In terms of macro and micronutrients, soils have sufficient K and Fe contents, high Ca and Mn contents, moderate Mg, Zn and Cu contents and highly low P contents (Table 6).

Although ideal vineyard soils are described in the literature as loamy (L) or sandy–loamy (SL), slightly gravelly, well-aerated, humus-rich and moderately calcareous, with the most suitable pH reported to be between 6 and 8 [16,71,74], the soil at the present site is, by contrast, a clayey, moderately alkaline (pH 8.02), highly calcareous (17.74% lime), low-organic-matter (0.92%) soil, conditions representative of the semi-arid viticultural region under study.

**Figure 5:**
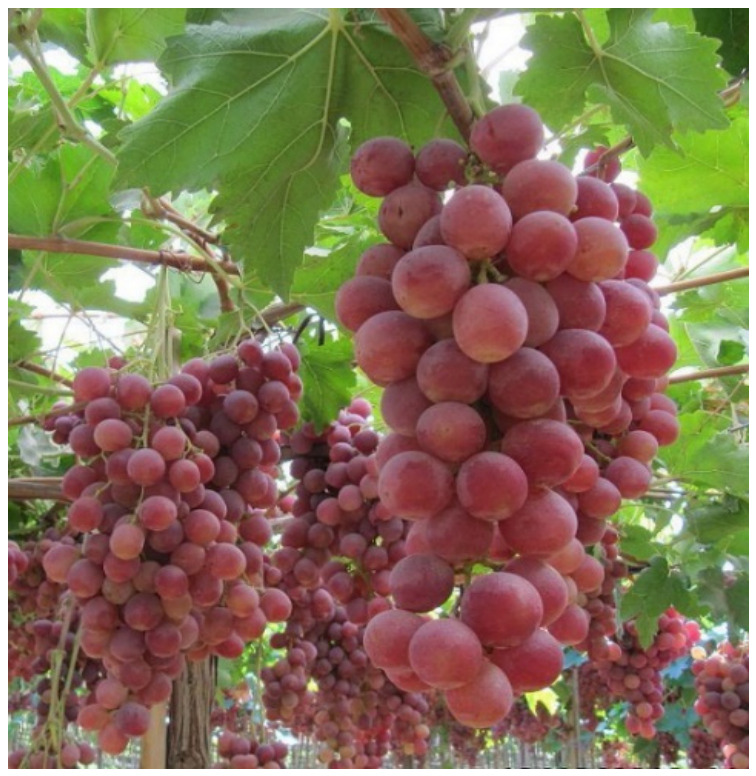
Red Globe grape variety (photograph; original).

General characteristics of plant material: The Red Globe grape variety (*Vitis vinifera* L.) is a hybrid of (Hunisa x Emperor) x Nocera varieties. The clusters are large–very large (1000 g) and plump (Figure 5). Berries are purplish-red in color and very large (12–14 g), round in shape and have 3–4 seeds. It is a variety that needs to be pruned short. It is a mid-season ripening variety [75].

Characteristics of micronized calcite (Multigreen = Herbagreen): The product is a chemically inert, 100% natural micronized calcite (calcium carbonate), marketed as Multigreen in Türkiye and as Herbagreen elsewhere (manufacturer: 5 K Mineral Ltd. Şti., Sarıçam, Adana, Türkiye). According to the manufacturer’s technical specifications, it has a particle-size range of 0.16 µm to 0.36 mm, a guaranteed total CaO content of 35% (*w*/*w*), a maximum moisture content of 20%, and a pH of 8–10; it is certified for organic agriculture (EKOTAR, TR-OT-006). These figures are reported for reproducibility and not as evidence of efficacy. It is produced using ‘mobile ionic technology’, a patented technology of calcite. The reduced particle size of this product is reported by the manufacturer as a technical specification. Under appropriate temperature conditions, maximum photosynthesis levels are achieved at approximately 0.1% CO_2_ with sufficient nutrient and water supplements. The product is marketed under the trade name Multigreen in Türkiye and is equivalent to Herbagreen, which has been evaluated in scientific studies on grapevines [29,30].

### 3.2. Experimental Design and Treatments

In the experimental vineyard, annual maintenance operations such as pruning, soil cultivation, fertilization and pesticide control were carried out under the control of the researcher.

During winter pruning, the vines were charged equally (20 ± 2 buds/vine). Irrigation was carried out periodically using drip irrigation system. All treatments received identical basal nutrition, applied uniformly through the drip irrigation system (fertigation) over the growing season and comprising 5.125 kg/da N, 5.75 kg/da P and 5.41 kg/da K as elemental nutrients (equivalent to 51.25, 57.5 and 54.1 kg/ha, respectively). Irrigation was applied through the drip system every four days at 2000 L/da. Plant protection followed a standard regional program of six sprays applied uniformly to all plots over the season, targeting dead-arm, powdery and downy mildew, gray mold, grape berry moth and mealybug (full schedule, active ingredients, doses and timings are given in Supplementary Materials. Consequently, any differences among treatments are attributable to the foliar micronized calcite applications rather than to background management.

Experimental design;

Control → 0.0% concentration

1st treatment (before flowering) → 0.5% concentration

2nd treatment (before flowering + after flowering) → 0.5 + 0.5% concentration

3rd treatment (before flowering + after flowering + véraison) → 0.5 + 0.5 + 0.5% concentration.

In present study, 60 vines [4 treatments (control, 1st, 2nd and 3rd treatments) × 3 replicates × 5 vines per replicate] were used. Each replication consisted of 7 vines. To eliminate the edge effect, the first and last vines in each replicate were considered border (buffer) plants, and data were collected only from the middle 5 vines.

### 3.3. Measurements and Analyses

Phenological observations: Phenological observations of the grapevines were determined by taking into account the classification of vine phenological stages made by Eichorn and Lorenz [76].

Climate data: The data were collected with the use of HOBO and Pyranometer devices from budburst to leaf fall date and relevant calculations were made accordingly.

Leaf analysis: Leaf analysis was conducted on 12 samples from control and three treatments (separately for each three repetitions) at Science and Technology Application and Research Center of Siirt University and the analyses were evaluated in accordance with Kacar and İnal [67].

Yield and quality analysis: Grape yield, number of clusters, cluster weight, cluster length, cluster width, berry length, berry width, 100-berry weight [77] and berry skin color [64] parameters were examined. Estimated yield per hectare (ton/ha) was calculated by multiplying the average yield per vine by the total number of vines per hectare (2222 vines/ha based on the 3.0 m × 1.5 m spacing).

Grape juice (must) composition analysis: Here, “must” denotes the freshly pressed, unfermented juice of the berries. Total acidity, pH, specific gravity [51], soluble solids content [52], ripening index [78,79], must yield [80], total phenolics [81,82] and total flavonoids [83] were analyzed. The must samples were filtered through filter paper prior to analysis to remove pulp and skin fragments. The ripening (maturity) index was calculated as the ratio of soluble solids content (°Brix) to titratable acidity expressed in g of tartaric acid per 100 mL of must (i.e., MI = °Brix ÷ [g tartaric acid/100 mL]), following the convention adopted in [38,39]. Total acidity values reported in g/L throughout the manuscript were converted to g/100 mL prior to this calculation. Pycnometric specific gravity was determined on the filtered must following the procedure described in [51], using a pycnometer calibrated with distilled water at 20 °C. The raw specific gravity data were re-examined and confirmed; no correction was required, and the values are presented in Figure 2. Specific gravity did not differ significantly among treatments (*p* ≥ 0.05).

### 3.4. Statistical Analysis

The experiment was conducted as a completely randomized design with four treatments (control, 1st, 2nd, and 3rd), three replicates per treatment, and five vines per replicate. The mean of the five vines per replicate served as the experimental unit (*n* = 3 per treatment), preserving the independence of observations. Normality of residuals and homogeneity of variances were verified prior to analysis using the Shapiro–Wilk and Levene’s tests, respectively. The data were subjected to one-way analysis of variance (ANOVA) using the Fit Model platform of SAS JMP 8 (SAS Institute Inc., Cary, NC, USA), and treatment means were compared using Tukey’s Honestly Significant Difference (HSD) test at α = 0.05 [84]. Tukey’s HSD was selected because it controls the family-wise Type I error rate across all pairwise contrasts. In each table, means not connected by a common letter differ significantly, and the corresponding HSD critical differences are reported. Non-significant differences (*p* ≥ 0.05) are listed in the tables for completeness but are not interpreted as treatment effects in the text. The coefficient of variation (VC) was calculated for each parameter as a further indicator of experimental reliability. With three replicates per treatment (*n* = 3), the statistical power to detect small-to-moderate effects is limited; non-significant numerical differences (*p* ≥ 0.05) should therefore be regarded as inconclusive rather than as evidence of no effect and are not interpreted as treatment effects in the text.

## 4. Conclusions

In the present single-season trial conducted under the semi-arid ecological conditions of Siirt province, Türkiye, foliar treatments of micronized calcite (Multigreen) were associated with measurable improvements in the yield and quality parameters of the Red Globe grape variety. Among the treatments evaluated, the single pre-bloom application (0.5%) was associated with the highest values for yield components (the number of clusters per vine, cluster weight, and total yield per vine exceeded the control by 48.67%, 51.16%, and 121%, respectively), as well as for must quality indices such as soluble solids content and ripening index. The double treatment (pre- and post-bloom) showed the highest values for physical berry dimensions, whereas the triple treatment showed the highest values for certain color parameters. These outcomes should be interpreted as preliminary, given the single-year nature of the dataset.

To our knowledge, this study represents one of the first systematic comparisons of stage-specific micronized calcite application strategies across a comprehensive set of yield, fruit quality, berry, and color parameters, supplemented by descriptive leaf nutrient characterization, in the Red Globe variety under semi-arid field conditions. Within the limits of this single-season experiment, the findings suggest that pre-bloom timing may represent a particularly responsive window for the biostimulant effect of micronized calcite in table grape production, a hypothesis that warrants confirmation through multi-year studies.

Several limitations of the present study should be explicitly acknowledged when interpreting these results. First, and most importantly, the data were collected during a single growing season (2022), which inherently restricts the robustness and generalizability of the findings, particularly under semi-arid conditions, where inter-annual variability in temperature, radiation, and water availability is well documented to substantially modulate vine physiology and yield response. Multi-year replication is therefore essential before the observed treatment effects can be considered reproducible or extrapolated to other vintages and locations. Second, the observed improvements in yield and quality parameters are interpreted here in the context of the well-established stimulatory role of calcium-based foliar products reported in the literature; however, the precise physiological pathways through which micronized calcite exerts these effects were not directly measured in this work and represent an important avenue for future investigation. Third, although percentage differences between treatments are reported to facilitate interpretation, only statistically significant differences should be treated as evidence of treatment effects, and non-significant trends should be regarded with caution. Accordingly, the conclusions drawn here should be considered preliminary and context-specific rather than broadly prescriptive. Fourth, because a single concentration (0.5%) was evaluated, application timing and application rate are confounded in the present design; it is therefore not possible to determine which factor primarily drives efficacy, and dose × timing factorial trials are required to resolve this.

Future research incorporating multi-year and multi-location trials, direct physiological measurements such as gas exchange and chlorophyll fluorescence, and comparative evaluations against other biostimulants will further advance the understanding of micronized calcite as a viticultural input. The present findings provide valuable field-level evidence supporting the single pre-bloom micronized calcite treatment as a practical and promising supplementary foliar fertilization strategy in semi-arid viticulture, contributing to the growing body of knowledge on sustainable crop management practices. From a practical standpoint, the single pre-bloom application requires only about one-third of the product, labor, and water inputs of the triple scheme while being associated with the highest yield response, suggesting the most favorable indicative cost–benefit ratio among the strategies tested; a formal economic analysis under local input and output prices is recommended as a next step.

## Figures and Tables

**Table 1:** Total effective temperature (TET) and solar radiation values.

	Total Effective Temperature (dd)				Solar Radiation (W/m^2^)			
Periods	Control	1st Treatments *	2nd Treatments **	3rd Treatments ***	Control	1st Treatments *	2nd Treatments **	3rd Treatments ***
Budburst—full bloom	347.52	313.75	313.75	313.75	227.55	238.81	238.81	238.81
Full Bloom—véraison	880.17	783.98	703.96	703.96	94.74	95.83	95.25	95.25
Véraison—harvest	1040.95	1169.30	1249.49	1249.49	76.21	77.33	79.03	79.03
Budburst—véraison	1215.71	1084.94	1004.92	1004.92	150.35	155.54	158.45	158.45
Full Bloom—harvest	1898.37	1932.95	1932.95	1932.95	86.04	86.10	86.10	86.10
Budburst—harvest ****	2233.91	2233.91	2233.91	2233.91	124.81	124.81	124.81	124.81
Budburst—senescence	3280.43	3278.69	3282.96	3284.52	114.21	114.36	114.79	114.75

* The 1st treatment was performed once before flowering (28.04.2022). ** The 2nd treatment was performed twice, before flowering (28.04.2022) and after flowering (25.05.2022). *** The 3rd treatment was performed three times, before flowering (28.04.2022), after flowering (25.05.2022) and after véraison (02.07.2022). **** Since harvest was practiced in the same day to compare the effects of treatments, TET and solar radiation values for budburst–harvest period were the same for all treatments.

**Table 2:** Effects of micronized calcite treatments on cluster, berry and leaf parameters.

Treatments	Cluster Width (cm)	Cluster Length (cm)	Berry Width (mm)	Berry Length (mm)	100-Berry Weight (g)	Number of Seeds per Berry	Average Single Seed Weight (gr)	Leaf Chlorophyll Content	Average Single Leaf Weight (gr)
Control	14.73 ± 0.97 b	21.96 ± 1.78	20.99 ± 0.63	21.69 ± 0.61	597.33 ± 71.94	3.33 ± 0.27	0.04 ± 0.00	41.18 ± 1.24	2.48 ± 0.42 b
1st treatments	18.19 ± 0.67 ab	21.08 ± 0.91	20.55 ± 0.77	21.88 ± 0.62	629.33 ± 4.91	3.07 ± 0.03	0.05 ± 0.00	43.03 ± 1.77	3.23 ± 0.23 ab
2nd treatments	19.89 ± 1.29 a	23.35 ± 0.15	21.50 ± 0.15	22.15 ± 0.06	663.67 ± 18.89	3.40 ± 0.12	0.05 ± 0.01	41.30 ± 0.85	3.90 ± 0.25 a
3rd treatments	18.99 ± 0.87 ab	21.53 ± 0.51	20.92 ± 0.41	21.98 ± 0.46	663.67 ± 29.24	3.30 ± 0.15	0.03 ± 0.00	42.94 ± 0.72	4.06 ± 0.07 a
Tukey HSD	4.419 *	4.683	2.461	2.231	181.311	0.747	0.018	5.505	1.245 *
VC	5.29	3.81	2.33	1.99	4.89	4.34	7.76	2.72	7.17

* *p* < 0.05. VC: coefficient of variation. Means within a column not connected by a common letter differ significantly according to Tukey’s HSD post hoc test (α = 0.05). ± indicates the standard error.

**Table 3:** Monthly temperature, relative humidity and solar radiation data of the experimental vineyard.

Months	Temperature (°C)			Relative Humidity (%)			Solar Radiation (W/m^2^) ***		
	Mean	Min.	Max.	Mean	Min.	Max.	Mean	Min.	Max.
April (05–30.04.2022) *	17.90	2.74	33.50	53.74	10.21	99.11	278.06	3.05	1001.20
May	24.16	8.89	40.29	37.19	9.68	97.27	106.71	3.05	894.40
June	28.29	12.82	44.38	25.55	5.86	57.42	92.53	3.05	924.90
July	32.66	18.53	46.48	25.05	7.74	53.58	78.70	3.05	149.55
August	30.80	17.56	43.89	26.95	8.18	67.50	77.48	3.05	384.60
September	24.49	9.04	40.14	33.52	8.58	97.03	122.51	3.05	827.25
October	18.27	4.43	35.66	42.56	8.97	98.35	63.19	3.05	271.65
November (01–25.11.2022) **	11.96	0.33	26.67	72.46	25.83	100.00	114.33	3.05	680.70

* Since budburst data was from 05.04.2022, records were initiated from this date. ** Since the latest senescence data was from 25.11.2022, records were terminated at this date. *** Since there was no solar radiation at nighttime, night data were not taken into consideration while calculating monthly average solar radiation and minimum solar radiation quantities.

**Table 4:** Leaf macro- and micronutrient contents under the experimental treatments (means ± SE; *n* = 3).

Treatments	B (ppm)	Ca (%)	Cu (ppm)	Fe (ppm)	K (%)	Mg (%)	Mn (ppm)	P (%)	Zn (ppm)	N (%)
Control	135.43 ± 11.83	1.51 ± 0.05	15.74 ± 0.19	233.73 ± 20.67	0.70 ± 0.04	0.51 ± 0.03	73.58 ± 0.72	0.17 ± 0.01	54.43 ± 6.52	3.11 ± 0.14
1st Treatments	121.31 ± 12.23	1.56 ± 0.04	15.43 ± 0.13	197.37 ± 9.51	0.81 ± 0.08	0.49 ± 0.05	76.92 ± 12.81	0.17 ± 0.00	56.65 ± 10.38	2.34 ± 0.21
2nd Treatments	134.97 ± 9.09	1.61 ± 0.16	15.69 ± 0.21	218.43 ± 31.96	0.58 ± 0.08	0.54 ± 0.02	101.01 ± 15.26	0.20 ± 0.02	64.28 ± 11.69	2.46 ± 0.62
3rd Treatments	121.73 ± 3.89	1.62 ± 0.15	15.78 ± 0.02	207.37 ± 16.78	0.60 ± 0.11	0.45 ± 0.02	73.71 ± 3.90	0.17 ± 0.01	60.90 ± 5.85	2.29 ± 0.35

Values are presented as means ± standard error of three replicates (*n* = 3). Data were subjected to one-way ANOVA followed by Tukey’s HSD test (α = 0.05); no statistically significant differences among treatments were detected for any nutrient (*p* ≥ 0.05). ANOVA *p*-values: B = 0.61, Ca = 0.91, Cu = 0.43, Fe = 0.67, K = 0.26, Mg = 0.29, Mn = 0.24, P = 0.21, Zn = 0.87, N = 0.44.

**Table 5:** Soil characteristics of the experimental vineyard.

CLAY(C) (%)	SILT(SI) (%)	SAND(S) (%)	Texture	pH	Salinity (dS/m)	Organic Matter (%)	Lime (%)
56.3	30.7	13.0	Clayey	8.02	0.17	0.92	17.74

**Table 6:** Macro and micronutrients of experimental vineyard.

Cu (ppm)	Mn (ppm)	Fe (ppm)	Zn (ppm)	B (ppm)	K_2_O (kg/da)	P_2_O_5_ (kg/da)	Ca (mg/kg)	Mg (mg/kg)
0.73	7.22	8.51	0.73	0.40	86.06	2.50	1882.87	347.56

## Data Availability

The data to support the results and conclusions of this study is presented within the article. Detailed data is available upon request.

## Supplementary Materials

The following supporting information can be downloaded at: https://www.mdpi.com/article/10.3390/plants15132013/s1, Table S1: Effects of micronized calcite treatments on yield parameters of Red Globe grapevines (data underlying Figure 1). Table S2: Effects of micronized calcite treatments on grape juice (must) composition of Red Globe grapevines (data underlying Figure 2). Table S3: Effects of micronized calcite treatments on berry color parameters (CIELAB) of Red Globe grapevines (data underlying Figure 3).
