# Supplementary material for: Micronized Calcite Foliar Treatments as an Approach to Enhancing Yield and Quality Parameters of Red Globe Grape (Vitis vinifera L.) Under Semi-Arid Conditions
Source: Plants (Basel). 2026 Jun 29;15(13):2013. doi: 10.3390/plants15132013 (PMC13364388; doi:10.3390/plants15132013)
Supplement: Supplementary file 1 [file plants-15-02013-s001.zip › plants-4380620-Supplementary.pdf]

Supplementary Tables S1–S3. Numerical data underlying Figures 1–3

These tables provide the full descriptive and inferential statistics (means ± standard error, Tukey HSD critical values, and coefficients of variation) for the parameters presented graphically in Figures 1–3 of the main text.

Supplementary Table S1. Effects of micronized calcite treatments on yield parameters of Red Globe grapevines (data underlying Figure 1).

TREATMENTS	Clusters per vine	Clusters per ha	Cluster weight (g)	Grape yield (kg/vine)
**Control**	20.63±1.20 b	45,851.4±266.15 b	419.27±0.02 c	8.72±0.20 c
**1st**	30.67±1.45 a	68,147.5±322.88 a	633.75±0.04 a	19.26±0.41 a
**2nd**	21.53±1.03 b	47,839.0±229.89 b	481.65±0.01 bc	10.54±0.19 c
**3rd**	24.00±0.53 b	53,332.8±117.59 b	590.25±0.02 ab	14.43±0.74 b
**Tukey HSD**	5.010***	1113.400***	0.111***	2.011***
**VC (%)**	4.35	4.35	4.16	2.91

*Values are means ± standard error (n = 3). Means within a column not sharing a common letter differ significantly (Tukey HSD, α = 0.05). VC: coefficient of variation. *** p < 0.001. Clusters per ha = clusters per vine × planting density (2223 vines/ha).*

Supplementary Table S2. Effects of micronized calcite treatments on grape juice (must) composition of Red Globe grapevines (data underlying Figure 2).

TREATMENTS	pH	SSC (%)	Maturity index	Must yield (mL)	Specific gravity	Total phenolics (mg/mL)	Total flavonoids (μg/mL)	Total acidity (g/L)
**Control**	4.41±0.06 a	14.18±0.46	28.14±0.92	1530.00±25.98 ab	1.01±0.13	0.23±0.01	3.31±0.09	5.05±0.20 a
**1st**	4.16±0.09 ab	14.98±0.02	32.92±1.13	1647.50±46.70 a	1.18±0.15	0.21±0.01	3.04±0.12	4.56±0.16 a
**2nd**	4.07±0.07 b	13.30±0.76	40.69±6.51	1505.00±51.30 ab	1.22±0.23	0.21±0.02	3.15±0.08	3.38±0.35 b
**3rd**	4.12±0.03 ab	13.00±0.58	38.32±3.63	1372.50±60.16 b	1.29±0.01	0.21±0.02	3.00±0.18	3.43±0.18 b
**Tukey HSD**	0.290*	2.392	17.195	216.086*	0.692	0.062	0.566	1.073**
**VC (%)**	1.44	3.26	8.70	3.04	10.36	6.33	3.79	5.46

*Values are means ± standard error (n = 3). Means within a column not sharing a common letter differ significantly (Tukey HSD, α = 0.05). VC: coefficient of variation. * p < 0.05, ** p < 0.01. Total phenolics expressed as gallic acid equivalent; total flavonoids as rutin equivalent; total acidity as tartaric acid equivalent.*

Supplementary Table S3. Effects of micronized calcite treatments on berry color parameters (CIELAB) of Red Globe grapevines (data underlying Figure 3).

TREATMENTS	L*	a*	b*	Chroma (C*)	Hue angle (h°)
**Control**	22.34±0.70	-0.89±0.17	2.75±0.28 ab	2.90±0.30 ab	107.75±2.73
**1st**	22.13±2.93	-1.04±0.28	3.11±0.49 a	3.28±0.56 a	107.91±1.84
**2nd**	19.64±1.00	-0.46±0.06	1.57±0.21 b	1.64±0.19 b	106.88±3.36
**3rd**	23.56±1.85	-1.19±0.09	3.90±0.24 a	4.08±0.25 a	106.99±0.77
**Tukey HSD**	8.322	0.780	1.471**	1.598**	10.793
**VC (%)**	7.39	-16.76	10.76	10.90	2.03

*Values are means ± standard error (n = 3). Means within a column not sharing a common letter differ significantly (Tukey HSD, α = 0.05). VC: coefficient of variation. ** p < 0.01. CIELAB: L* = 0 black (dark) to 100 white (light); a* = +60 red to −60 green; b* = +60 yellow to −60 blue.*
